# Defining Incidental Versus Non-incidental COVID-19 Hospitalizations

**DOI:** 10.7759/cureus.56546

**Published:** 2024-03-20

**Authors:** Dhimitri A Nikolla, Jonathan J Oskvarek, Mark S Zocchi, Nishad A Rahman, Andrew Leubitz, Ali Moghtaderi, Bernard S Black, Jesse M Pines

**Affiliations:** 1 Department of Internal Medicine / Emergency Medicine, Lake Erie College of Osteopathic Medicine, Erie, USA; 2 Department of Emergency Medicine, Allegheny Health Network, Erie, USA; 3 US Acute Care Solutions (USACS) Research Group, US Acute Care Solutions, Canton, USA; 4 Department of Emergency Medicine, Summa Health System, Akron, USA; 5 Heller School for Social Policy and Management, Brandeis University, Waltham, USA; 6 Department of Emergency Medicine, LifeBridge Health - Sinai Hospital, Baltimore, USA; 7 Department of Emergency Medicine, Adventist HealthCare - Shady Grove Medical Center, Rockville, USA; 8 Milken Institute School of Public Health, George Washington University, Washington, DC, USA; 9 Pritzker School of Law and Kellogg School of Management, Northwestern University, Chicago, USA; 10 Department of Emergency Medicine, Allegheny Health Network, Pittsburgh, USA

**Keywords:** dexamethasone, hospital admission, health policy, public health, internal medicine, emergency medicine, pandemic, coronavirus disease 2019

## Abstract

Background

Rates of COVID-19 hospitalization are an important measure of the health system burden of severe COVID-19 disease and have been closely followed throughout the pandemic. The highly transmittable, but often less severe, Omicron COVID-19 variant has led to an increase in hospitalizations with incidental COVID-19 diagnoses where COVID-19 is not the primary reason for admission. There is a strong public health need for a measure that is implementable at low cost with standard electronic health record (EHR) datasets that can separate these incidental hospitalizations from non-incidental hospitalizations where COVID-19 is the primary cause or an important contributor. Two crude metrics are in common use. The first uses in-hospital administration of dexamethasone as a marker of non-incidental COVID-19 hospitalizations. The second, used by the United States (US) CDC, relies on a limited set of COVID-19-related diagnoses (i.e., respiratory failure, pneumonia). Both measures likely undercount non-incidental COVID-19 hospitalizations. We therefore developed an improved EHR-based measure that is better able to capture the full range of COVID-19 hospitalizations.

Methods

We conducted a retrospective study of ED visit data from a national emergency medicine group from April 2020 to August 2023. We assessed the CDC approach, the dexamethasone-based measure, and alternative approaches that rely on co-diagnoses likely to be related to COVID-19, to determine the proportion of non-incidental COVID-19 hospitalizations.

Results

Of the 153,325 patients diagnosed with COVID-19 at 112 general EDs in 17 US states, and admitted or transferred, our preferred measure classified 108,243 (70.6%) as non-incidental, compared to 71,066 (46.3%) using the dexamethasone measure and 77,399 (50.5%) using the CDC measure.

Conclusions

Identifying non-incidental COVID-19 hospitalizations using ED administration of dexamethasone or the CDC measure provides substantially lower estimates than our preferred measure.

## Introduction

Background

Since the start of the Coronavirus disease - 2019 (COVID-19) pandemic, national and local governments and healthcare organizations have struggled to monitor the pandemic’s severity and the degree of burden on healthcare resources [1]. In an era of widespread at-home testing [2], infection counts have become increasingly unreliable and are often not reported [3-7]. Therefore, COVID-19 hospitalizations remain an important measure of the COVID-19 burden. However, starting in December 2021, the dominance of the highly infectious but often less severe Omicron variant led to increasing numbers of hospitalized patients who tested positive for COVID-19 but were admitted primarily for other reasons [8,9]. This rise in hospitalizations of patients with incidental COVID-19 infections blurs the signal provided by hospitalization counts for the COVID-19 burden on the population and hospitals. 

Importance

Despite the importance of hospitalizations as a measure of the public health burden of COVID-19, there is no good measure that can reliably distinguish between cases where COVID-19 is the primary reason for hospitalization (non-incidental admission) and cases where COVID-19 is incidental to the hospitalization. As we discuss below, the two principal measures in current use undercount non-incidental hospitalizations, especially during the Omicron period that began in December 2021. 

One approach, developed by the Commonwealth of Massachusetts, uses the in-hospital administration of dexamethasone as a proxy for non-incidental COVID-19 hospitalizations [10-11]. Dexamethasone is a steroid medication indicated for patients with COVID-19-associated acute respiratory failure with hypoxemia [12]. Yet in the Omicron era, patients hospitalized with non-incidental COVID-19 are less likely to have severe respiratory failure with hypoxemia [13,14]. In our experience as ED clinicians, many COVID-19-positive patients, especially older patients, are admitted to hospitals (or transferred for admission) with COVID-19 symptoms and complications but without an indication for dexamethasone. Similarly, some patients may have a contraindication to receiving dexamethasone, such as an allergy. Therefore, relying solely on dexamethasone to count non-incidental COVID-19 hospitalizations likely lacks sensitivity as a measure of which patients with COVID-19 are hospitalized primarily for their COVID-19 infection. The dexamethasone proxy also has limited specificity because dexamethasone may be prescribed for conditions not due to COVID-19 infection. For example, dexamethasone may be administered for allergic reactions, pretreatment for IV contrast administration to allergic patients, or adrenal insufficiency.

The second method in common use to identify non-incidental COVID-19 hospitalizations relies on a limited number of co-diagnoses identified by the CDC as being related to COVID-19 [15,16]. The CDC has reported several iterations of these highly related co-diagnoses [16]. During the early pandemic, the CDC developed a COVID-19 hospitalization dashboard which indicated how often COVID-19-related discharge diagnoses included co-diagnoses for acute respiratory distress syndrome, acute respiratory failure, pneumonia, sepsis, and acute renal failure/kidney injury [15,16]. Since these diagnoses were available at the conception of our study, we modeled our CDC definition on these diagnoses. Nevertheless, all iterations of diagnoses used by the CDC to identify non-incidental COVID-19 hospitalizations rely heavily on a co-diagnosis of acute respiratory failure [15,16]. Thus, the definition is highly correlated with the dexamethasone measure and has similar shortcomings. Therefore, the CDC approach also likely undercounts non-incidental COVID-19 hospitalizations, especially during the Omicron period.

Study objective

We sought to develop an improved method for measuring the proportion of COVID-19-positive patients admitted/transferred from the ED for non-incidental COVID-19, which could be applied using diagnoses that should be available either from the ED visit alone (as we use it), from the hospitalization alone, or from both together. Conceptually, these and other COVID-19-related diagnoses (e.g., acute metabolic encephalopathy) would often also be present at the time of admission to the ED [17]. We hypothesized that relying on a set of co-diagnoses that are strongly related to COVID-19 infection, but a broader set than the CDC has used, can provide a reasonable way to identify non-incidental COVID-19 hospitalizations. Conversely, we did not view dexamethasone administration as adding materially to the information already available for a patient with COVID-19 and a diagnosis of respiratory failure with hypoxemia, the indication for which dexamethasone is recommended. 

Practical consideration: need for a measure that can be simply and cheaply used at scale

Prior studies using manual chart review have estimated non-incidental COVID-19 admissions to be between 55-88% of all hospitalizations of COVID-19-positive patients pre-Omicron and 69% after the onset of Omicron [18-22]. However, manual chart reviews are impracticable at scale. The public health need is for a simpler measure that can be implemented relying on information already captured in standard EHRs. We therefore present an approach to measuring the approximate incidence of non-incidental COVID-19 hospitalizations using COVID-19-related co-diagnose codes.

## Materials and methods

Study design, setting, and patients

We conducted a retrospective study of billing and visit data from a national emergency medicine physician group, in which we studied patients who visited the ED, what proportion were diagnosed with COVID-19, of those, what proportion were either admitted or transferred to another facility, and the co-diagnoses for these patients. Transfers were counted together with admissions because admission to another hospital is the most common reason for transfer from the ED to another facility [23]. Below we refer to admitted or transferred patients simply as admitted. The dataset has been described previously [24,25]. Briefly, charts are reviewed by billing and coding specialists shortly after the ED visit. These specialists have ongoing training and undergo regular quality and compliance audits to ensure accurate coding of diagnoses using International Classification of Disease, Tenth Revision (ICD-10) codes. Visit information, including primary and secondary diagnoses, procedures, and medications administered during the ED stay are stored in a de-identified research dataset. Our final study dataset included all visits to 112 non-pediatric, non-freestanding EDs (in 17 states) with data for the full study period from April 1, 2020, to August 31, 2023.

We collected ED visit dates, medication orders for dexamethasone, primary ED diagnoses, secondary ED diagnoses (up to two), and ED disposition (i.e., admitted, transferred, discharged, left without being seen) for each encounter in the study period. Since dexamethasone administration is used by others to measure non-incidental COVID-19 hospitalizations [10-11], visits with missing medication data (2.6% of all visits) were excluded. We selected patients with ED-diagnosed COVID-19 using the Agency for Healthcare Research and Quality Clinical Classification Software Refined (CCSR) code INF012 [26]. We examined the proportions of admitted COVID-19 patients hospitalized specifically for symptoms or complications of COVID-19 based on various definitions, including the dexamethasone definition, the CDC definition, and the definition we developed and how these proportions varied over the study period. The Allegheny Health Network Institutional Review Board approved secondary analyses of this de-identified research dataset.

Variables

We compared several approaches for determining which patients were admitted for non-incidental COVID-19 versus those admitted with an incidental COVID-19 infection. We considered a dexamethasone-only approach, using dexamethasone administration to identify non-incidental COVID-19 hospitalizations [11]. We also considered the CDC approach, classifying COVID-19 hospitalizations with concomitant acute renal failure/acute kidney injury, acute respiratory distress syndrome, acute respiratory failure, pneumonia, and sepsis as non-incidental [15]. However, both the dexamethasone-based and CDC definitions will, based on our clinical experience, miss admissions for conditions that are often consequences of, or associated with, a COVID-19 infection, for example, those with acute metabolic encephalopathy or acute pulmonary embolism [17,27,28].

We therefore sought to develop a more sensitive definition based on co-diagnoses that are likely to indicate a non-incidental admission. As a basis for choosing a set of co-diagnoses that are likely to indicate a non-incidental admission, we reviewed all Agency for Healthcare Research and Quality (AHRQ) Clinical Classifications Software Refined (CCSR) codes (N=543, excluding the INF012 CCSR code for COVID-19) and divided them into categories signifying the strength of their association with COVID-19 [26]. Two board-certified ED-physician authors (JO and DN) independently marked each CCSR code “Maybe Related” or “Not Related” with a third ED-physician author (JP) resolving disagreements. Then, three authors (JO, NR, and DN) independently marked all the “Maybe Related” CCSR codes as “High Likelihood,” “Medium Likelihood,” or “Low Likelihood.” Agreement between two of the three raters decided the final classification, except for a small number of codes (N=4) for which all three raters disagreed, which we considered “Medium Likelihood.” Classifications and details on interrater reliability are presented in the appendix. We classified 23 CCSR codes as High Likelihood, 34 as Medium Likelihood, and 14 as Low Likelihood. The remaining 472 CCSR codes were considered Not Related.

We then used these categories to identify COVID-19-related diagnoses and define approaches to measuring non-incidental COVID-19 admissions. The approaches included: 1) an upper bound approach including all high and medium likelihood diagnoses, 2) a diagnosis-based approach including all high likelihood diagnoses, 3) a mixed, high-likelihood diagnosis or dexamethasone approach, including all patients with high likelihood diagnoses or with dexamethasone ordered in the ED, 4) a CDC-based approach adopted on the CDC’s list of COVID-19 associated diagnoses [15], which corresponds to the CCSR categories for pneumonia, septicemia, respiratory failure, or acute renal failure, and 5) a dexamethasone-only approach where dexamethasone was ordered in the ED. ED-diagnosed COVID-19 patients admitted without any secondary diagnoses were counted as non-incidental COVID-19 admissions for all approaches except dexamethasone-only.

Recall, however, that dexamethasone for the treatment of COVID-19 is indicated when acute respiratory failure with hypoxemia is present [12]. The diagnosis-based approach, which includes all diagnosis variants of respiratory failure (Table 1), should capture all of these cases. Therefore, there should be little difference between approach 2 (high-likelihood diagnoses only) and approach 3 (high-likelihood or dexamethasone). Moreover, any admissions captured by approach 3 but not approach 2 would have to involve cases where dexamethasone was administered in the absence of the indication for its use for COVID-19 patients. Thus, these incremental cases can be considered false positives, where dexamethasone was administered for an indication other than acute respiratory failure with hypoxemia.

**Table 1:** Approaches to defining admissions/transfers for COVID-19 CCSR, Clinical Classifications Software Refined; CDC, Centers for Disease Control and Prevention ED patients admitted/transferred with a single diagnosis within the CCSR code INF012 for COVID-19 (no secondary diagnoses) were counted as non-incidental COVID-19 admissions for all approaches except dexamethasone-only. See appendix for CCSR codes designated as related to COVID-19 or not.

Approach	Definition
Upper bound	The patient had a diagnosis categorized as Maybe COVID-Related – High or Medium Likelihood.
Diagnosis-based or dexamethasone	The patient had a diagnosis categorized as Maybe Related – High Likelihood OR a dexamethasone order.
Diagnosis-based	The patient had a diagnosis categorized as Maybe Related – High Likelihood.
CDC-based	The patient had a diagnosis under the following CCSR codes: RSP002, INF002, RSP012, or GEN002.
Dexamethasone-Only	The patient had a dexamethasone order.

Outcomes

The principal study outcomes were the proportion of all admitted COVID-19 ED patients having non-incidental COVID-19 classified using the defined approaches. Since the severity of COVID-19 illness and the dominant virus variants have changed over time [7,13,14], the proportions of non-incidental admissions were plotted by month over the study period to examine trends.

Analysis

Categorical and continuous variables are presented as counts with percentages and means with standard deviations (SD). Since the Omicron variant of COVID-19 often causes less severe respiratory illness [13,14], we studied the five approaches separately before and after the onset of Omicron. The pre-Omicron (April 1, 2020, to December 18, 2021) and Omicron (December 19, 2021, to August 31, 2023) periods are based on metadata associated with sequences available on GISAID, and accessible at doi.org/10.55876/gis8.220330me [29]. Since dexamethasone may be prescribed and administered either in the ED or hospital, we compared the proportion of non-incidental COVID-19 hospitalizations using this study’s ED-based dexamethasone-only approach to the proportion of COVID-19 hospitalizations with dexamethasone administration reported by the Commonwealth of Massachusetts in 2022 using inpatient data [30]. Interrater reliability for classifying COVID-19-related diagnoses was measured using Fleiss' Kappa (see Appendix). The analysis was conducted with Stata v.17 (StataCorp LLC, College Station, Texas, United States) and R version 4.2.1 (R Foundation for Statistical Computing, Vienna, Austria).

## Results

**Figure 1:**
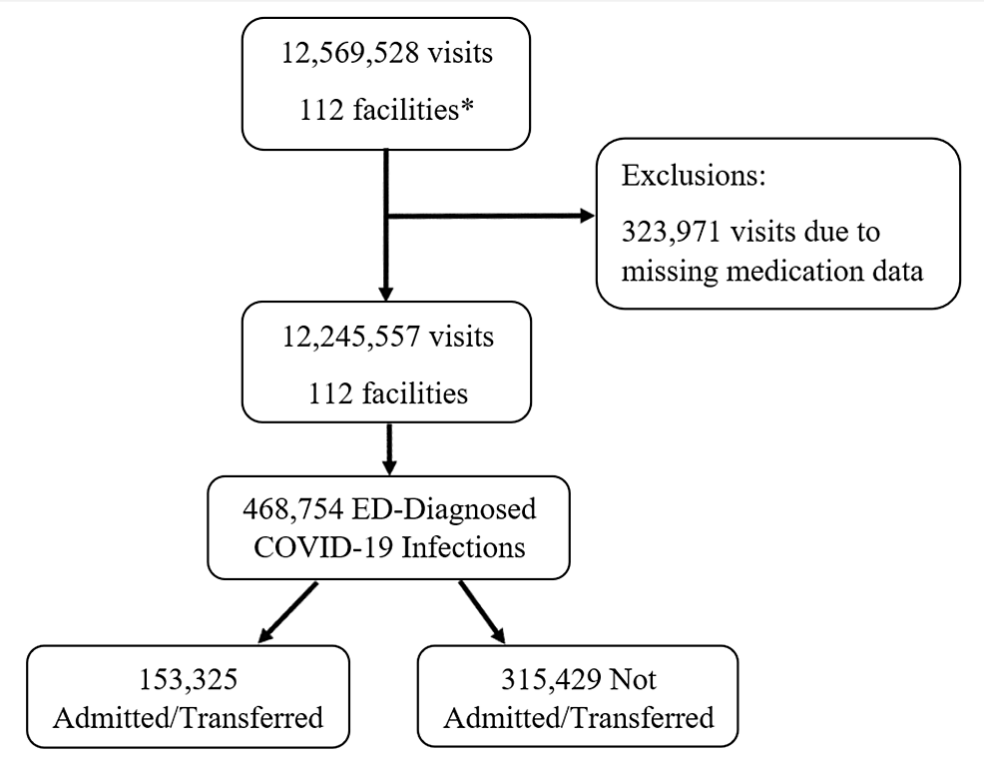
Study flow diagram depicting the study population and exclusions. *General emergency departments (i.e., not pediatric, not freestanding) with a full panel (42 months) of data.

Of the 12,569,528 ED visits to 112 facilities in 17 US states during the study period, 323,971 visits were excluded for missing medication data (Figure 1, Table 2). Of the remaining visits, 468,754 visits included a positive COVID-19 diagnosis; of these, 153,325 were admitted (Figure 1). The mean age in years of admitted COVID-19 positive patients during the study period was 64 (SD 18.6), including 62 (17.8) pre-Omicron and 67 (19.5) after the onset of Omicron (Table 3). Co-diagnoses of pneumonia decreased from 39.5% to 17.9% and respiratory failure from 20.1% to 14.7% between the pre-Omicron and Omicron periods (Table 3).

**Table 2:** Facility characteristics. ^a^Five sites in this study were not with the physician group in 2019.

Characteristics	N	(%)
Facility total visits in 2019		
<30k	40	(35.7)
30k-59,999	52	(46.4)
60k and over	15	(13.4)
Unknown^a^	5	(4.5)
Facility metro status		
Non-metro	30	(26.8)
Metro	82	(73.2)

**Table 3:** Demographics for ED patients admitted/transferred with a COVID-19 diagnosis. SD, standard deviation ^a^The Pre-Omicron period is from April 1, 2020, to December 18, 2021. The Omicron period is from December 19, 2021, to August 31, 2023 (end of available study data). The delineation between periods is based on metadata associated with sequences available on Global Initiative on Sharing All Influenza Data (GISAID), and accessible at doi.org/10.55876/gis8.220330me. ^b^Diagnoses under CCSR codes: RSP002, INF002, RSP012, and GEN002.

	Pre-Omicron^a^		Omicron^a^		Total	
	N	(%)	N	(%)	N	(%)
Patient age (years)						
<10	374	(0.4)	991	(1.8)	1,365	(0.9)
10-17	375	(0.4)	288	(0.5)	663	(0.4)
18-54	29,524	(30.3)	10,269	(18.4)	39,793	(26.0)
55 and over	67,050	(68.9)	44,392	(79.4)	111,442	(72.7)
Patient age, mean (SD)	62	(17.8)	67	(19.5)	64	(18.6)
Selected co-diagnoses^b^						
Pneumonia	38,414	(39.5)	10,027	(17.9)	48,441	(31.6)
Sepsis	5,848	(6.0)	4,027	(7.2)	9,875	(6.4)
Respiratory failure	19,574	(20.1)	8,250	(14.7)	27,824	(18.1)
Acute renal failure	4,714	(4.8)	3,502	(6.3)	8,216	(5.4)
Female patients	46,336	(47.6)	28,209	(50.4)	74,545	(48.6)
Payer source						
Medicare	47,181	(48.5)	35,682	(63.8)	82,863	(54.1)
Medicaid	9,992	(10.3)	6,360	(11.4)	16,352	(10.7)
Commercial	25,814	(26.5)	8,432	(15.1)	34,246	(22.3)
Self-pay	11,832	(12.2)	4,075	(7.3)	15,907	(10.4)
Other	2,517	(2.6)	1,408	(2.5)	3,925	(2.6)

**Figure 2:**
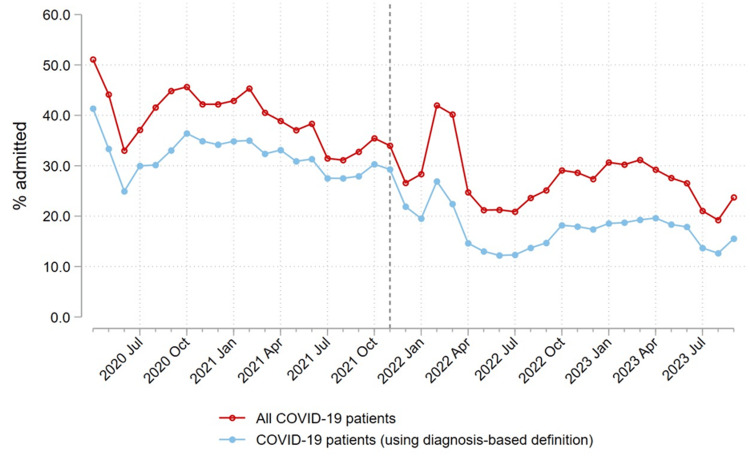
Total percent of COVID-19 infections admitted/transferred. Line plots of the percentage of ED patients with COVID-19 infection who were admitted/transferred over the study period (denominator being all COVID-19-infected ED patients), including all infected patients and those in the diagnosis-based definition.

**Figure 3:**
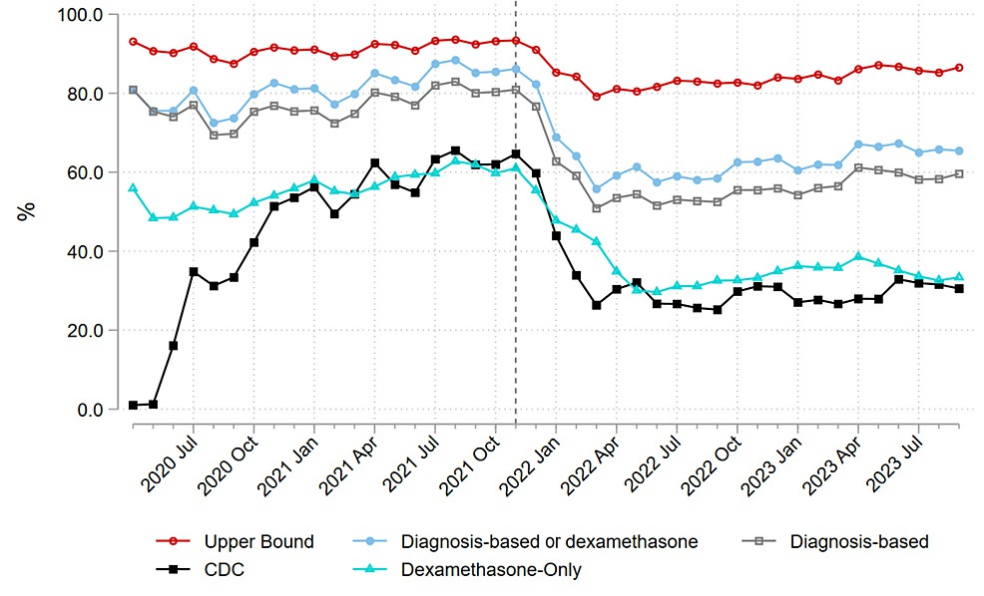
Percent of COVID-19 admissions admitted for COVID-19. Line plot of the percentage of admissions/transfers for COVID-19 over the study period using different definitions of non-incidental COVID-19.

The proportion of ED-diagnosed COVID-19 patients admitted decreased over the study period (Figure 2). The proportion of patients admitted with non-incidental COVID-19 was roughly flat during the pre-Omicron period for all approaches except the dexamethasone-only approach and fell with all approaches at the onset of the Omicron period and then flattened out again (Figure 3). The proportion of non-incidental COVID-19 admissions varied greatly between measurement approaches (Figure 3 and Table 4). The proportion of patients admitted for COVID-19 over the study period was as high as 89.0% using the upper bound approach (averaged over the sample period) or as low as 50.5% with the dexamethasone-only approach (Figure 3 and Table 4). In 2020, before dexamethasone became the standard of care for COVID-19-associated acute respiratory failure with hypoxemia, the CDC-based approach estimated more non-incidental COVID-19 hospitalizations than the dexamethasone-only approach. However, in 2021 and 2022, the two approaches yielded similar estimates (Figure 3 and Table 4). The number of probable false positive cases captured by the dexamethasone-only definition but not by the high-likelihood diagnosis approach was estimated to be 4,773 of 97,323 (4.9%) for the pre-Omicron period, 3,439 of 55,940 (6.1%) for the Omicron period, and 8,212 of 153,263 (5.4%) over the study period (Table 4).

**Table 4:** COVID-19 patients admitted/transferred for non-incidental COVID-19. ^a^The Pre-Omicron period is from April 1, 2020, to December 18, 2021. The Omicron period is from December 19, 2021, to August 31, 2023, (end of available study data). ^b^Since the diagnosis-based approach includes acute hypoxemic respiratory failure with hypoxemia and other respiratory failure diagnoses, the additional cases captured by the diagnosis-based or dexamethasone approach are likely false positive cases with dexamethasone administered for indications other than COVID-19.

	Pre-Omicron^a^		Omicron^a^		Total	
Approach	No.	%	No.	%	No.	%
All admitted/transferred	97,323		55,940		153,263	
Upper bound	89,316	(91.7)	47,095	(84.1)	136,411	(89.0)
Diagnosis-based or dexamethasone^b^	80,471	(82.7)	35,984	(64.3)	116,455	(76.0)
Diagnosis-based^b^	75,698	(77.8)	32,545	(58.1)	108,243	(70.6)
CDC-based	51,925	(53.3)	19,141	(34.2)	71,066	(46.3)
Dexamethasone-only	55,679	(57.2)	21,720	(38.8)	77,399	(50.5)

**Figure 4:**
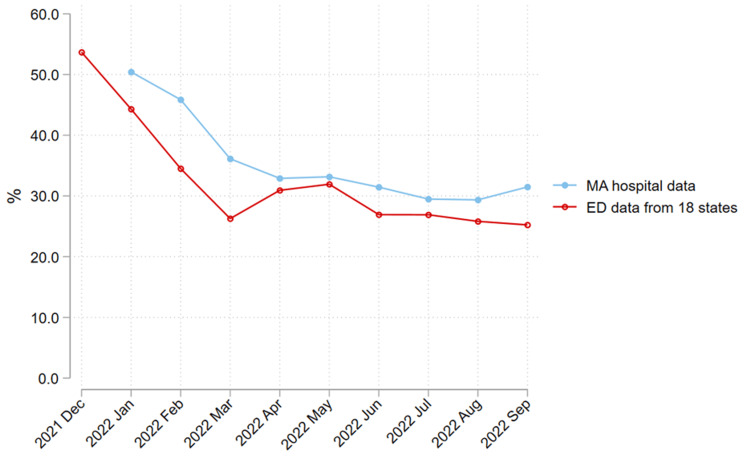
Percent of hospitalized COVID-19 patients receiving dexamethasone. Line graph of the percent of COVID-19 hospitalizations receiving dexamethasone over time comparing Massachusetts (MA) in-hospital data to US Acute Care Solutions (USACS) national ED data.

The use of dexamethasone decreased over the study period across multiple subgroups. For example, the percentage of patients receiving dexamethasone in the ED decreased between the pre-Omicron and Omicron periods from 65.7% to 57.0% for those diagnosed with pneumonia, 49.1% to 33.9% with sepsis, 74.7% to 67.8% with respiratory failure, and 42.0% to 27.1% with acute renal failure (Table 5). The lower dexamethasone-only proportions, relative to the co-diagnosis approaches, are unlikely to be due to differences between the ED-based counts we used and the in-hospital counts used by Massachusetts. From January 10, 2022, to September 27, 2022, levels and time trends were similar for our approach versus Massachusetts’ publicly reported data (Figure 4) [30].

**Table 5:** Percentage of admissions/transfers with COVID-19-related co-diagnoses that received dexamethasone in the ED. ^a^Diagnoses under CCSR codes: RSP002, INF002, RSP012, and GEN002.

	Pre-Omicron		Omicron		Overall	
	Received dexamethasone	Total	Received dexamethasone	Total	Received dexamethasone	Total
Co-diagnosis^a^	N (%)	N	N (%)	N	N (%)	N (%)
Pneumonia	25,225 (65.7)	38,414	5,713 (57.0)	10,027	30,938 (63.9)	48,441
Sepsis	2,869 (49.1)	5,848	1,367 (33.9)	4,027	4,236 (42.9)	9,875
Respiratory failure	14,631 (74.7)	19,574	5,594 (67.8)	8,250	20,225 (72.7)	27,824
Acute renal failure	1,978 (42.0)	4,714	950 (27.1)	3,502	2,928 (35.6)	8,216

## Discussion

In our multisite study, the diagnosis-based approach developed by our study team to distinguish non-incidental COVID-19 hospitalizations from incidental ones estimated many more non-incidental COVID-19 hospitalizations than the CDC-based approach or the dexamethasone approach (Figure 3 and Table 4), and a proportion of non-incidental admissions similar to estimates based on chart reviews, as discussed below. Our clinical assessment is that the diagnosis-based approach yields a more accurate estimate of non-incidental COVID-19 hospitalizations than the CDC-based or dexamethasone approaches. In particular, in our experience, patients hospitalized for COVID-19 can have various non-pulmonary sequelae, and many do not meet the clinical indications for dexamethasone administration. Therefore, the CDC-based and dexamethasone approaches, which yield similar results, are likely to substantially undercount the number of non-incidental COVID-19 hospitalizations (Table 4), especially given the reduced incidence and often reduced severity of pneumonia and respiratory failure after the onset of Omicron (Table 4 and Table 5) [13,14]. This undercounting would underestimate the actual COVID-19 related burden on the population, and on hospitals and health systems.

However, the upper bound approach may overcount hospitalizations. This potential overcounting is illustrated by the less drastic drop in non-incidental hospitalizations at the onset of Omicron (Figure 3). The more gradual decline with the upper bound approach compared to the other approaches can be explained by more COVID-19 infected patients being hospitalized with less-related co-diagnoses (Table 1).

We also showed that while the dexamethasone approach undercounts non-incidental hospitalizations, it also counts a substantial number of false positives. We estimated that 5.4% of admitted COVID-19 ED patients could be falsely classified as non-incidental by the dexamethasone approach during the study period (Table 4). This finding was expected given that dexamethasone has many indications other than respiratory failure with hypoxemia due to COVID-19 infection (e.g., allergic reactions).

Additionally, despite having similar estimates and trends (Figure 3 and Table 4), we observed evidence that the dexamethasone and CDC-based approaches likely count different patients. For example, in the study period, only a fraction of patients with pneumonia (63.9%), sepsis (42.9%), respiratory failure (72.7%), and acute renal failure (35.6%) received dexamethasone during the study period (Table 5). If the dexamethasone and CDC-based approaches counted the same patients, these percentages would more closely approximate 100% given the similar estimates both approaches provide for non-incidental COVID-19 hospitalizations. 

Our results align with prior studies estimating the proportion of non-incidental COVID-19 hospitalizations using chart review methods. For example, McAlister et al. trained research assistants to perform chart reviews to classify the proportion of non-incidental COVID-19 hospitalizations from March 2020 to July 2022 for 14,290 cases using primary and secondary hospital discharge diagnoses [9]. They reported that 70% of COVID-19 hospitalizations were directly caused by COVID-19 [9]. Similarly, Klann et al. had clinical experts perform chart reviews from March 2020 to March 2021 on 1,123 randomly sampled COVID-19 hospitalizations using diagnoses and symptoms from clinical notes and lab results to classify non-incidental COVID-19 hospitalizations [19]. They reported that 68% of COVID-19 hospitalizations were admitted for non-incidental COVID-19 infection [19]. Other studies using chart review methods have estimated similar proportions of non-incidental COVID-19 hospitalizations, 55-88% [18,20-22]. These results align with our diagnosis-based approach, which estimates non-incidental COVID-19 hospitalizations at 70.6% on average over the sample period although generally below 60% from April 2022 through the end of the sample period in August 2023 (Figure 3 and Table 4). Our approach was also sensitive enough to capture an apparent uptick in the proportion of non-incidental admissions from around 55% to 60% in April 2023, coinciding with the expiration of the US public health emergency authorizations. Nevertheless, our diagnosis-based approach can be implemented at scale without labor-intensive chart review.

We also observed a similar proportion of non-incidental COVID-19 hospitalizations using our ED-based dexamethasone approach as was reported by Massachusetts using hospitalization data in the Omicron period (Figure 4) [30]. This suggests that the difference between measuring ED-based administration of dexamethasone (our study) and hospital-based administration (Massachusetts reporting), does not explain the lower rates of non-incidental COVID-19 hospitalizations we found using the dexamethasone approach.

Systematic undercounting of non-incidental COVID-19 hospitalizations can have important public health implications. First, COVID-19 hospitalization estimates are used to make public health and policy decisions impacting large segments of the population. These health and policy decisions, such as implementing mask and vaccine mandates, may have economic and educational consequences (e.g., reduced employment rates, reduced child test scores, etc.) [31]. In addition, the estimates may change individuals’ behaviors by affecting their perception of the COVID-19 risk they face. Therefore, since dexamethasone has a limited indication for acute respiratory failure with hypoxemia and dexamethasone use has been decreasing with the Omicron variant (Table 5), we believe that better methods to estimate the proportions of incidental and non-incidental COVID-19 hospitalizations are available. The CDC approach performed similarly to the dexamethasone approach. To correct the likely undercounting from both approaches, we propose the diagnosis-based approach as an alternative with greater face validity (Table 1, Figure 3).

Limitations

Our study was retrospective, observational, and limited to variables that were systematically collected by the emergency medicine group, such as ICD-10 diagnoses and medication orders. Using different variables than the ones we chose, or a manual chart review, may result in different estimates of non-incidental COVID-19 hospitalizations. Future research might examine integrating more clinical information (e.g., vital signs) into the definitions identifying non-incidental COVID-19 infections. Furthermore, there is no gold standard criteria for comparison to classify incidental versus non-incidental COVID-19 hospitalizations. For example, the CDC has had multiple iterations of its diagnosis-based definition [16]. Also, we did not capture ED diagnoses beyond the first three diagnoses (primary plus two secondary). We also used CCSR codes, rather than assessing relevant co-diagnoses directly from ICD-10 codes. Lastly, the interrater reliability between raters in choosing high-, medium-, and low-likelihood co-diagnoses was fair. Therefore, a different set of raters, or non-ED clinicians, may have classified the CCSR codes differently. Finally, our results were limited to ED visits during the study period and may not generalize to new variants, which may have different disease manifestations.

## Conclusions

We propose an approach to measuring which hospitalizations of patients with ED-diagnosed COVID-19 infections were non-incidental based on co-diagnoses that are highly related to COVID-19 infection. This approach counts substantially more non-incidental hospitalizations than the dexamethasone or CDC-based approaches and comports better with our clinical experience as ED physicians who must decide which COVID-19-positive patients to admit and with the available evidence from chart-review-based studies. 

## Appendices

Interrater reliability details for CCSR code relationship to COVID-19 categories for diagnosis approaches

After excluding the INF012 CCSR code for COVID-19, Fleiss’ Kappa was 0.803 (z = 18.1, p-value < 0.01, n = 543) between the two raters (JO and DN), determining which CCSR codes were “Maybe Related” or “Not Related” to COVID-19. Between the three raters (JO, NR, and DN) determining which “Maybe Related” CCSR codes were “High Likelihood,” “Medium Likelihood,” or “Low Likelihood,” Fleiss’ Kappa was 0.221 (z = 4.47, p-value < 0.01, n = 71).

Given the high interrater reliability between “Maybe Related” and “Not Related”, we also assessed interrater reliability for only “High Likelihood” and “High/Medium Likelihood” diagnoses between the three raters (JO, NR, and DN), assuming they agreed on which diagnoses were “Not Related” (Table 6). For determining which diagnoses were “High Likelihood” or not between the three raters, Fleiss’ Kappa would be 0.546 (z = 22, p-value < 0.01, n = 543). For determining which diagnoses were “High/Medium Likelihood” or not between the three raters, Fleiss’ Kappa would be 0.832 (z = 33.6, p-value < 0.01, n = 543).

**Table 6:** Clinical Classifications Software Refined (CCSR) codes used to classify diagnoses as related to COVID-19 or not. Clinical Classifications Software Refined (CCSR) codes group diagnosis codes into similar clinically meaningful categories [26].

Category	Code	Description
High Likelihood	CIR018	Cardiac arrest and ventricular fibrillation
High Likelihood	CIR031	Hypotension
High Likelihood	FAC016	Exposure, encounters, screening, or contact with infectious disease
High Likelihood	INF008	Viral infection
High Likelihood	INF011	Sequela of specified infectious disease conditions
High Likelihood	NVS010	Headache; including migraine
High Likelihood	NVS018	Myopathies
High Likelihood	PNL005	Respiratory distress syndrome
High Likelihood	RSP001	Sinusitis
High Likelihood	RSP002	Pneumonia (except that caused by tuberculosis)
High Likelihood	RSP005	Acute bronchitis
High Likelihood	RSP006	Other specified upper respiratory infections
High Likelihood	RSP007	Other specified and unspecified upper respiratory disease
High Likelihood	RSP008	Chronic obstructive pulmonary disease and bronchiectasis
High Likelihood	RSP009	Asthma
High Likelihood	RSP012	Respiratory failure; insufficiency; arrest
High Likelihood	RSP016	Other specified and unspecified lower respiratory disease
High Likelihood	SYM002	Fever
High Likelihood	SYM003	Shock
High Likelihood	SYM004	Nausea and vomiting
High Likelihood	SYM007	Malaise and fatigue
High Likelihood	SYM012	Circulatory signs and symptoms
High Likelihood	SYM013	Respiratory signs and symptoms
Medium Likelihood	BLD006	Coagulation and hemorrhagic disorders
Medium Likelihood	BLD010	Other specified and unspecified hematologic conditions
Medium Likelihood	CIR005	Myocarditis and cardiomyopathy
Medium Likelihood	CIR006	Pericarditis and pericardial disease
Medium Likelihood	CIR009	Acute myocardial infarction
Medium Likelihood	CIR012	Nonspecific chest pain
Medium Likelihood	CIR013	Acute pulmonary embolism
Medium Likelihood	CIR015	Other and ill-defined heart disease
Medium Likelihood	CIR016	Conduction disorders
Medium Likelihood	CIR017	Cardiac dysrhythmias
Medium Likelihood	CIR019	Heart failure
Medium Likelihood	CIR020	Cerebral infarction
Medium Likelihood	CIR023	Occlusion or stenosis of precerebral or cerebral arteries without infarction
Medium Likelihood	CIR025	Sequela of cerebral infarction and other cerebrovascular disease
Medium Likelihood	CIR026	Peripheral and visceral vascular disease
Medium Likelihood	CIR030	Aortic and peripheral arterial embolism or thrombosis
Medium Likelihood	CIR032	Other specified and unspecified circulatory disease
Medium Likelihood	CIR033	Acute phlebitis, thrombophlebitis, and thromboembolism
Medium Likelihood	CIR039	Other specified diseases of veins and lymphatics
Medium Likelihood	DIG001	Intestinal infection
Medium Likelihood	DIG007	Gastritis and duodenitis
Medium Likelihood	DIG008	Other specified and unspecified disorders of stomach and duodenum
Medium Likelihood	END011	Fluid and electrolyte disorders
Medium Likelihood	GEN002	Acute and unspecified renal failure
Medium Likelihood	INF002	Septicemia
Medium Likelihood	MUS036	Autoinflammatory syndromes
Medium Likelihood	NVS013	Coma, stupor, and brain damage
Medium Likelihood	RSP004	Acute and chronic tonsillitis
Medium Likelihood	RSP010	Aspiration pneumonitis
Medium Likelihood	RSP011	Pleurisy, pleural effusion, and pulmonary collapse
Medium Likelihood	SYM005	Dysphagia
Medium Likelihood	SYM006	Abdominal pain and other digestive/abdomen signs and symptoms
Medium Likelihood	SYM016	Other general signs and symptoms
Medium Likelihood	SYM017	Abnormal findings without diagnosis
Low Likelihood	CIR010	Complications of acute myocardial infarction
Low Likelihood	CIR011	Coronary atherosclerosis and other heart disease
Low Likelihood	CIR024	Other and ill-defined cerebrovascular disease
Low Likelihood	CIR034	Chronic phlebitis, thrombophlebitis, and thromboembolism
Low Likelihood	DIG018	Hepatic failure
Low Likelihood	DIG019	Other specified and unspecified liver disease
Low Likelihood	FAC017	No immunization or underimmunization
Low Likelihood	FAC024	Carrier status
Low Likelihood	INF003	Bacterial infections
Low Likelihood	INF004	Fungal infections
Low Likelihood	INF007	Hepatitis
Low Likelihood	INJ076	Complication, sequela
Low Likelihood	NVS009	Epilepsy, convulsions
Low Likelihood	NVS012	Transient cerebral ischemia
Not Related	BLD001	Nutritional anemia
Not Related	BLD002	Hemolytic anemia
Not Related	BLD003	Aplastic anemia
Not Related	BLD004	Acute posthemorrhagic anemia
Not Related	BLD005	Sickle cell trait/anemia
Not Related	BLD007	Diseases of white blood cells
Not Related	BLD008	Immunity disorders
Not Related	BLD009	Postprocedural or postoperative complications of the spleen
Not Related	CIR001	Chronic rheumatic heart disease
Not Related	CIR002	Acute rheumatic heart disease
Not Related	CIR003	Nonrheumatic and unspecified valve disorders
Not Related	CIR004	Endocarditis and endocardial disease
Not Related	CIR007	Essential hypertension
Not Related	CIR008	Hypertension with complications and secondary hypertension
Not Related	CIR014	Pulmonary heart disease
Not Related	CIR021	Acute hemorrhagic cerebrovascular disease
Not Related	CIR022	Sequela of hemorrhagic cerebrovascular disease
Not Related	CIR027	Arterial dissections
Not Related	CIR028	Gangrene
Not Related	CIR029	Aortic, peripheral, and visceral artery aneurysms
Not Related	CIR035	Varicose veins of lower extremity
Not Related	CIR036	Postthrombotic syndrome and venous insufficiency/hypertension
Not Related	CIR037	Vasculitis
Not Related	CIR038	Postprocedural or postoperative circulatory system complication
Not Related	DIG002	Disorders of teeth and gingiva
Not Related	DIG003	Diseases of the mouth excluding dental
Not Related	DIG004	Esophageal disorders
Not Related	DIG005	Gastroduodenal ulcer
Not Related	DIG006	Gastrointestinal and biliary perforation
Not Related	DIG009	Appendicitis and other appendiceal conditions
Not Related	DIG010	Abdominal hernia
Not Related	DIG011	Regional enteritis and ulcerative colitis
Not Related	DIG012	Intestinal obstruction and ileus
Not Related	DIG013	Diverticulosis and diverticulitis
Not Related	DIG014	Hemorrhoids
Not Related	DIG015	Anal and rectal conditions
Not Related	DIG016	Peritonitis and intra-abdominal abscess
Not Related	DIG017	Biliary tract disease
Not Related	DIG020	Pancreatic disorders (excluding diabetes)
Not Related	DIG021	Gastrointestinal hemorrhage
Not Related	DIG022	Noninfectious gastroenteritis
Not Related	DIG023	Noninfectious hepatitis
Not Related	DIG024	Postprocedural or postoperative digestive system complication
Not Related	DIG025	Other specified and unspecified gastrointestinal disorders
Not Related	EAR001	Otitis media
Not Related	EAR002	Diseases of the middle ear and mastoid (except otitis media)
Not Related	EAR003	Diseases of the inner ear and related conditions
Not Related	EAR004	Hearing loss
Not Related	EAR005	Postprocedural or postoperative ear and/or mastoid process complication
Not Related	EAR006	Other specified and unspecified disorders of the ear
Not Related	END001	Thyroid disorders
Not Related	END002	Diabetes mellitus without complication
Not Related	END003	Diabetes mellitus with complication
Not Related	END004	Diabetes mellitus, Type 1
Not Related	END005	Diabetes mellitus, Type 2
Not Related	END006	Diabetes mellitus due to underlying condition, drug or chemical induced, or other specified type
Not Related	END007	Nutritional deficiencies
Not Related	END008	Malnutrition
Not Related	END009	Obesity
Not Related	END010	Disorders of lipid metabolism
Not Related	END012	Cystic fibrosis
Not Related	END013	Pituitary disorders
Not Related	END014	Postprocedural or postoperative endocrine or metabolic complication
Not Related	END015	Other specified and unspecified endocrine disorders
Not Related	END016	Other specified and unspecified nutritional and metabolic disorders
Not Related	END017	Sequela of malnutrition and other nutritional deficiencies
Not Related	EXT001	External cause codes: cut/pierce, initial encounter
Not Related	EXT002	External cause codes: drowning/submersion, initial encounter
Not Related	EXT003	External cause codes: fall, initial encounter
Not Related	EXT004	External cause codes: fire/burn, initial encounter
Not Related	EXT005	External cause codes: firearm, initial encounter
Not Related	EXT006	External cause codes: machinery, initial encounter
Not Related	EXT007	External cause codes: motor vehicle traffic (MVT), initial encounter
Not Related	EXT008	External cause codes: pedal cyclist; not MVT, initial encounter
Not Related	EXT009	External cause codes: pedestrian; not MVT, initial encounter
Not Related	EXT010	External cause codes: transport; not MVT, initial encounter
Not Related	EXT011	External cause codes: natural/environment, initial encounter
Not Related	EXT012	External cause codes: bites, initial encounter
Not Related	EXT013	External cause codes: overexertion, initial encounter
Not Related	EXT014	External cause codes: poisoning by drug
Not Related	EXT015	External cause codes: poisoning by non-drug
Not Related	EXT016	External cause codes: struck by, against, initial encounter
Not Related	EXT017	External cause codes: suffocation/inhalation, initial encounter
Not Related	EXT018	External cause codes: other specified, classifiable, and NEC, initial encounter
Not Related	EXT019	External cause codes: unspecified mechanism
Not Related	EXT020	External cause codes: intent of injury, accidental/unintentional
Not Related	EXT021	External cause codes: intent of injury, self-harm
Not Related	EXT022	External cause codes: intent of injury, assault
Not Related	EXT023	External cause codes: intent of injury, undetermined
Not Related	EXT024	External cause codes: intent of injury, legal intervention/war
Not Related	EXT025	External cause codes: complications of medical and surgical care, initial encounter
Not Related	EXT026	External cause codes: activity codes
Not Related	EXT027	External cause codes: place of occurrence of the external cause
Not Related	EXT028	External cause codes: evidence of alcohol involvement
Not Related	EXT029	External cause codes: subsequent encounter
Not Related	EXT030	External cause codes: sequela
Not Related	EYE001	Cornea and external disease
Not Related	EYE002	Cataracts and other lens disorders
Not Related	EYE003	Glaucoma
Not Related	EYE004	Uveitis and ocular inflammation
Not Related	EYE005	Retinal and vitreous conditions
Not Related	EYE006	Neuro-ophthalmology
Not Related	EYE007	Strabismus
Not Related	EYE008	Oculofacial plastics and orbital conditions
Not Related	EYE009	Refractive error
Not Related	EYE010	Blindness and vision defects
Not Related	EYE011	Postprocedural or postoperative eye complication
Not Related	EYE012	Other specified eye disorders
Not Related	FAC001	Encounter for administrative purposes
Not Related	FAC002	Encounter for mental health services related to abuse
Not Related	FAC003	Encounter for observation and examination for conditions ruled out (excludes infectious disease, neoplasm, mental disorders)
Not Related	FAC004	Encounter for prophylactic or other procedures
Not Related	FAC005	Encounter for prophylactic measures (excludes immunization)
Not Related	FAC006	Encounter for antineoplastic therapies
Not Related	FAC007	Encounter for mental health conditions
Not Related	FAC008	Neoplasm-related encounters
Not Related	FAC009	Implant, device, or graft-related encounter
Not Related	FAC010	Other aftercare encounter
Not Related	FAC011	Counseling related to sexual behavior or orientation
Not Related	FAC012	Other specified encounters and counseling
Not Related	FAC013	Contraceptive and procreative management
Not Related	FAC014	Medical examination/evaluation
Not Related	FAC015	Resistance to antimicrobial drugs
Not Related	FAC018	Screening for neurocognitive or neurodevelopmental condition
Not Related	FAC019	Socioeconomic/psychosocial factors
Not Related	FAC020	Lifestyle/life management factors
Not Related	FAC021	Personal/family history of disease
Not Related	FAC022	Acquired absence of limb or organ
Not Related	FAC023	Organ transplant status
Not Related	FAC025	Other specified status
Not Related	GEN001	Nephritis, nephrosis, and renal sclerosis
Not Related	GEN003	Chronic kidney disease
Not Related	GEN004	Urinary tract infections
Not Related	GEN005	Calculus of urinary tract
Not Related	GEN006	Other specified and unspecified diseases of the kidney and ureters
Not Related	GEN007	Other specified and unspecified diseases of the bladder and urethra
Not Related	GEN008	Urinary incontinence
Not Related	GEN009	Hematuria
Not Related	GEN010	Proteinuria
Not Related	GEN011	Vesicoureteral reflux
Not Related	GEN012	Hyperplasia of prostate
Not Related	GEN013	Inflammatory conditions of male genital organs
Not Related	GEN014	Erectile dysfunction
Not Related	GEN015	Male infertility
Not Related	GEN016	Other specified male genital disorders
Not Related	GEN017	Nonmalignant breast conditions
Not Related	GEN018	Inflammatory diseases of female pelvic organs
Not Related	GEN019	Endometriosis
Not Related	GEN020	Prolapse of female genital organs
Not Related	GEN021	Menstrual disorders
Not Related	GEN022	Benign ovarian cyst
Not Related	GEN023	Menopausal disorders
Not Related	GEN024	Female infertility
Not Related	GEN025	Other specified female genital disorders
Not Related	GEN026	Postprocedural or postoperative genitourinary system complication
Not Related	INF001	Tuberculosis
Not Related	INF005	Foodborne intoxications
Not Related	INF006	HIV infection
Not Related	INF009	Parasitic, other specified and unspecified infections
Not Related	INF010	Sexually transmitted infections (excluding HIV and hepatitis)
Not Related	INJ001	Fracture of head and neck, initial encounter
Not Related	INJ002	Fracture of the spine and back, initial encounter
Not Related	INJ003	Fracture of torso, initial encounter
Not Related	INJ004	Fracture of the upper limb, initial encounter
Not Related	INJ005	Fracture of the lower limb (except hip), initial encounter
Not Related	INJ006	Fracture of the neck of the femur (hip), initial encounter
Not Related	INJ007	Dislocations, initial encounter
Not Related	INJ008	Traumatic brain injury (TBI); concussion, initial encounter
Not Related	INJ009	Spinal cord injury (SCI), initial encounter
Not Related	INJ010	Internal organ injury, initial encounter
Not Related	INJ011	Open wounds of head and neck, initial encounter
Not Related	INJ012	Open wounds to limbs, initial encounter
Not Related	INJ013	Open wounds of the trunk, initial encounter
Not Related	INJ014	Amputation of a limb, initial encounter
Not Related	INJ015	Amputation of other body parts, initial encounter
Not Related	INJ016	Injury to blood vessels, initial encounter
Not Related	INJ017	Superficial injury; contusion, initial encounter
Not Related	INJ018	Crushing injury, initial encounter
Not Related	INJ019	Burn and corrosion, initial encounter
Not Related	INJ020	Effect of foreign body entering opening, initial encounter
Not Related	INJ021	Effect of other external causes, initial encounter
Not Related	INJ022	Poisoning by drugs, initial encounter
Not Related	INJ023	Toxic effects, initial encounter
Not Related	INJ024	Sprains and strains, initial encounter
Not Related	INJ025	Injury to nerves, muscles, and tendons, initial encounter
Not Related	INJ026	Other specified injury
Not Related	INJ027	Other unspecified injury
Not Related	INJ028	Adverse effects of drugs and medicaments, initial encounter
Not Related	INJ029	Underdosing of drugs and medicaments, initial encounter
Not Related	INJ030	Drug-induced or toxic-related condition
Not Related	INJ031	Allergic reactions
Not Related	INJ032	Maltreatment/abuse
Not Related	INJ033	Complication of cardiovascular device, implant or graft, initial encounter
Not Related	INJ034	Complication of genitourinary device, implant or graft, initial encounter
Not Related	INJ035	Complication of internal orthopedic device or implant, initial encounter
Not Related	INJ036	Complication of transplanted organs or tissue, initial encounter
Not Related	INJ037	Complication of other surgical or medical care, injury, initial encounter
Not Related	INJ038	Fracture of head and neck, subsequent encounter
Not Related	INJ039	Fracture of the spine and back, subsequent encounter
Not Related	INJ040	Fracture of torso, subsequent encounter
Not Related	INJ041	Fracture of the upper limb, subsequent encounter
Not Related	INJ042	Fracture of lower limb (except hip), subsequent encounter
Not Related	INJ043	Fracture of the neck of the femur (hip), subsequent encounter
Not Related	INJ044	Dislocations, subsequent encounter
Not Related	INJ045	Traumatic brain injury (TBI), concussion, subsequent encounter
Not Related	INJ046	Spinal cord injury (SCI), subsequent encounter
Not Related	INJ047	Internal organ injury, subsequent encounter
Not Related	INJ048	Open wounds of head and neck, subsequent encounter
Not Related	INJ049	Open wounds to limbs, subsequent encounter
Not Related	INJ050	Open wounds of the trunk, subsequent encounter
Not Related	INJ051	Amputation of a limb, subsequent encounter
Not Related	INJ052	Amputation of other body parts, subsequent encounter
Not Related	INJ053	Injury to blood vessels, subsequent encounter
Not Related	INJ054	Superficial injury; contusion, subsequent encounter
Not Related	INJ055	Crushing injury, subsequent encounter
Not Related	INJ056	Burns and corrosion, subsequent encounter
Not Related	INJ057	Effect of foreign body entering opening, subsequent encounter
Not Related	INJ058	Effect of other external causes, subsequent encounter
Not Related	INJ059	Poisoning by drugs, subsequent encounter
Not Related	INJ060	Toxic effects, subsequent encounter
Not Related	INJ061	Sprains and strains, subsequent encounter
Not Related	INJ062	Injury to nerves, muscles, and tendons, subsequent encounter
Not Related	INJ063	Other specified injury, subsequent encounter
Not Related	INJ064	Other unspecified injuries, subsequent encounter
Not Related	INJ065	Adverse effects of drugs and medicaments, subsequent encounter
Not Related	INJ066	Underdosing of drugs and medicaments, subsequent encounter
Not Related	INJ067	Allergic reactions, subsequent encounter
Not Related	INJ068	Maltreatment/abuse, subsequent encounter
Not Related	INJ069	Complication of cardiovascular device, implant or graft, subsequent encounter
Not Related	INJ070	Complication of genitourinary device, implant or graft, subsequent encounter
Not Related	INJ071	Complication of internal orthopedic device or implant, subsequent encounter
Not Related	INJ072	Complication of other surgical or medical care, injury, subsequent encounter
Not Related	INJ073	Injury, sequela
Not Related	INJ074	Effect of other external causes, sequela
Not Related	INJ075	Poisoning/toxic effect/adverse effects/underdosing, sequela
Not Related	MAL001	Cardiac and circulatory congenital anomalies
Not Related	MAL002	Digestive congenital anomalies
Not Related	MAL003	Genitourinary congenital anomalies
Not Related	MAL004	Nervous system congenital anomalies
Not Related	MAL005	Congenital malformations of eye, ear, face, neck
Not Related	MAL006	Cleft lip or palate
Not Related	MAL007	Respiratory congenital malformations
Not Related	MAL008	Musculoskeletal congenital conditions
Not Related	MAL009	Chromosomal abnormalities
Not Related	MAL010	Other specified and unspecified congenital anomalies
Not Related	MBD001	Schizophrenia spectrum and other psychotic disorders
Not Related	MBD002	Depressive disorders
Not Related	MBD003	Bipolar and related disorders
Not Related	MBD004	Other specified and unspecified mood disorders
Not Related	MBD005	Anxiety and fear-related disorders
Not Related	MBD006	Obsessive-compulsive and related disorders
Not Related	MBD007	Trauma- and stressor-related disorders
Not Related	MBD008	Disruptive, impulse-control, and conduct disorders
Not Related	MBD009	Personality disorders
Not Related	MBD010	Feeding and eating disorders
Not Related	MBD011	Somatic disorders
Not Related	MBD012	Suicidal ideation/attempt/intentional self-harm
Not Related	MBD013	Miscellaneous mental and behavioral disorders/conditions
Not Related	MBD014	Neurodevelopmental disorders
Not Related	MBD017	Alcohol-related disorders
Not Related	MBD018	Opioid-related disorders
Not Related	MBD019	Cannabis-related disorders
Not Related	MBD020	Sedative-related disorders
Not Related	MBD021	Stimulant-related disorders
Not Related	MBD022	Hallucinogen-related disorders
Not Related	MBD023	Inhalant-related disorders
Not Related	MBD024	Tobacco-related disorders
Not Related	MBD025	Other specified substance-related disorders
Not Related	MBD026	Mental and substance use disorders in remission
Not Related	MBD027	Suicide attempt/intentional self-harm, subsequent encounter
Not Related	MBD028	Opioid-related disorders, subsequent encounter
Not Related	MBD029	Stimulant-related disorders, subsequent encounter
Not Related	MBD030	Cannabis-related disorders, subsequent encounter
Not Related	MBD031	Hallucinogen-related disorders, subsequent encounter
Not Related	MBD032	Sedative-related disorders, subsequent encounter
Not Related	MBD033	Inhalant-related disorders, subsequent encounter
Not Related	MBD034	Mental and substance use disorders, sequela
Not Related	MUS001	Infective arthritis
Not Related	MUS002	Osteomyelitis
Not Related	MUS003	Rheumatoid arthritis and related disease
Not Related	MUS004	Juvenile arthritis
Not Related	MUS005	Other specified chronic arthropathy
Not Related	MUS006	Osteoarthritis
Not Related	MUS007	Other specified joint disorders
Not Related	MUS008	Immune-mediated/reactive arthropathies
Not Related	MUS009	Tendon and synovial disorders
Not Related	MUS010	Musculoskeletal pain, not low back pain
Not Related	MUS011	Spondylopathies/spondyloarthropathy (including infective)
Not Related	MUS012	Biomechanical lesions
Not Related	MUS013	Osteoporosis
Not Related	MUS014	Pathological fracture, initial encounter
Not Related	MUS015	Pathological fracture, subsequent encounter
Not Related	MUS016	Stress fracture, initial encounter
Not Related	MUS017	Stress fracture, subsequent encounter
Not Related	MUS018	Atypical fracture, initial encounter
Not Related	MUS019	Atypical fracture, subsequent encounter
Not Related	MUS020	Pathological, stress and atypical fractures, sequela
Not Related	MUS021	Acquired foot deformities
Not Related	MUS022	Scoliosis and other postural dorsopathy deformities
Not Related	MUS023	Acquired deformities (excluding foot)
Not Related	MUS024	Systemic lupus erythematosus and connective tissue disorders
Not Related	MUS025	Other specified connective tissue disease
Not Related	MUS026	Muscle disorders
Not Related	MUS027	Musculoskeletal abscess
Not Related	MUS028	Other specified bone diseases and musculoskeletal deformities
Not Related	MUS029	Disorders of jaw
Not Related	MUS030	Aseptic necrosis and osteonecrosis
Not Related	MUS031	Traumatic arthropathy
Not Related	MUS032	Neurogenic/neuropathic arthropathy
Not Related	MUS033	Gout
Not Related	MUS034	Crystal arthropathies (excluding gout)
Not Related	MUS035	Osteomalacia
Not Related	MUS037	Postprocedural or postoperative musculoskeletal system complication
Not Related	MUS038	Low back pain
Not Related	NEO001	Head and neck cancers - eye
Not Related	NEO002	Head and neck cancers - lip and oral cavity
Not Related	NEO003	Head and neck cancers - throat
Not Related	NEO004	Head and neck cancers - salivary gland
Not Related	NEO005	Head and neck cancers - nasopharyngeal
Not Related	NEO006	Head and neck cancers - hypopharyngeal
Not Related	NEO007	Head and neck cancers - pharyngeal
Not Related	NEO008	Head and neck cancers - laryngeal
Not Related	NEO009	Head and neck cancers - tonsils
Not Related	NEO010	Head and neck cancers - all other types
Not Related	NEO011	Cardiac cancers
Not Related	NEO012	Gastrointestinal cancers - esophagus
Not Related	NEO013	Gastrointestinal cancers - stomach
Not Related	NEO014	Gastrointestinal cancers - small intestine
Not Related	NEO015	Gastrointestinal cancers - colorectal
Not Related	NEO016	Gastrointestinal cancers - anus
Not Related	NEO017	Gastrointestinal cancers - liver
Not Related	NEO018	Gastrointestinal cancers - bile duct
Not Related	NEO019	Gastrointestinal cancers - gallbladder
Not Related	NEO020	Gastrointestinal cancers - peritoneum
Not Related	NEO021	Gastrointestinal cancers - all other types
Not Related	NEO022	Respiratory cancers
Not Related	NEO023	Bone cancer
Not Related	NEO024	Sarcoma
Not Related	NEO025	Skin cancers - melanoma
Not Related	NEO026	Skin cancers - basal cell carcinoma
Not Related	NEO027	Skin cancers - squamous cell carcinoma
Not Related	NEO028	Skin cancers - all other types
Not Related	NEO029	Breast cancer - ductal carcinoma in situ (DCIS)
Not Related	NEO030	Breast cancer - all other types
Not Related	NEO031	Female reproductive system cancers - uterus
Not Related	NEO032	Female reproductive system cancers - cervix
Not Related	NEO033	Female reproductive system cancers - ovary
Not Related	NEO034	Female reproductive system cancers - fallopian tube
Not Related	NEO035	Female reproductive system cancers - endometrium
Not Related	NEO036	Female reproductive system cancers - vulva
Not Related	NEO037	Female reproductive system cancers - vagina
Not Related	NEO038	Female reproductive system cancers - all other types
Not Related	NEO039	Male reproductive system cancers - prostate
Not Related	NEO040	Male reproductive system cancers - testis
Not Related	NEO041	Male reproductive system cancers - penis
Not Related	NEO042	Male reproductive system cancers - all other types
Not Related	NEO043	Urinary system cancers - bladder
Not Related	NEO044	Urinary system cancers - ureter and renal pelvis
Not Related	NEO045	Urinary system cancers - kidney
Not Related	NEO046	Urinary system cancers - urethra
Not Related	NEO047	Urinary system cancers - all other types
Not Related	NEO048	Nervous system cancers - brain
Not Related	NEO049	Nervous system cancers - all other types
Not Related	NEO050	Endocrine system cancers - thyroid
Not Related	NEO051	Endocrine system cancers - pancreas
Not Related	NEO052	Endocrine system cancers - thymus
Not Related	NEO053	Endocrine system cancers - adrenocortical
Not Related	NEO054	Endocrine system cancers - parathyroid
Not Related	NEO055	Endocrine system cancers - pituitary gland
Not Related	NEO056	Endocrine system cancers - all other types
Not Related	NEO057	Hodgkin lymphoma
Not Related	NEO058	Non-Hodgkin lymphoma
Not Related	NEO059	Leukemia - acute lymphoblastic leukemia (ALL)
Not Related	NEO060	Leukemia - acute myeloid leukemia (AML)
Not Related	NEO061	Leukemia - chronic lymphocytic leukemia (CLL)
Not Related	NEO062	Leukemia - chronic myeloid leukemia (CML)
Not Related	NEO063	Leukemia - hairy cell
Not Related	NEO064	Leukemia - all other types
Not Related	NEO065	Multiple myeloma
Not Related	NEO066	Malignant neuroendocrine tumors
Not Related	NEO067	Mesothelioma
Not Related	NEO068	Myelodysplastic syndrome (MDS)
Not Related	NEO069	Cancer of other sites
Not Related	NEO070	Secondary malignancies
Not Related	NEO071	Malignant neoplasm, unspecified
Not Related	NEO072	Neoplasms of unspecified nature or uncertain behavior
Not Related	NEO073	Benign neoplasms
Not Related	NEO074	Conditions due to neoplasm or the treatment of neoplasm
Not Related	NVS001	Meningitis
Not Related	NVS002	Encephalitis
Not Related	NVS003	Other specified central nervous system (CNS) infection and poliomyelitis
Not Related	NVS004	Parkinson`s disease
Not Related	NVS005	Multiple sclerosis
Not Related	NVS006	Other nervous system disorders (often hereditary or degenerative)
Not Related	NVS007	Cerebral palsy
Not Related	NVS008	Paralysis (other than cerebral palsy)
Not Related	NVS011	Neurocognitive disorders
Not Related	NVS014	CNS abscess
Not Related	NVS015	Polyneuropathies
Not Related	NVS016	Sleep-wake disorders
Not Related	NVS017	Nerve and nerve root disorders
Not Related	NVS019	Nervous system pain and pain syndromes
Not Related	NVS020	Other nervous system disorders (neither hereditary nor degenerative)
Not Related	NVS021	Postprocedural or postoperative nervous system complication
Not Related	NVS022	Sequela of specified nervous system conditions
Not Related	PNL001	Liveborn
Not Related	PNL002	Short gestation; low birth weight; and fetal growth retardation
Not Related	PNL003	Neonatal acidemia and hypoxia
Not Related	PNL004	Neonatal cerebral disorders
Not Related	PNL006	Respiratory perinatal condition
Not Related	PNL007	Hemolytic jaundice and perinatal jaundice
Not Related	PNL008	Birth trauma
Not Related	PNL009	Perinatal infections
Not Related	PNL010	Newborns affected by maternal conditions or complications of labor/delivery
Not Related	PNL011	Hemorrhagic and hematologic disorders of newborn
Not Related	PNL012	Neonatal digestive and feeding disorders
Not Related	PNL013	Other specified and unspecified perinatal conditions
Not Related	PNL014	Neonatal abstinence syndrome
Not Related	PNL015	Fetal alcohol syndrome
Not Related	PRG001	Antenatal screening
Not Related	PRG002	Gestational weeks
Not Related	PRG003	Spontaneous abortion and complications of spontaneous abortion
Not Related	PRG004	Induced abortion and complications of termination of pregnancy
Not Related	PRG005	Ectopic pregnancy and complications of ectopic pregnancy
Not Related	PRG006	Molar pregnancy and other abnormal products of conception
Not Related	PRG007	Complications following ectopic and/or molar pregnancy
Not Related	PRG008	Supervision of high-risk pregnancy
Not Related	PRG009	Early, first, or unspecified trimester hemorrhage
Not Related	PRG010	Hemorrhage after the first trimester
Not Related	PRG011	Early or threatened labor
Not Related	PRG012	Multiple gestations
Not Related	PRG013	Maternal care related to fetal conditions
Not Related	PRG014	Polyhydramnios and other problems of the amniotic cavity
Not Related	PRG015	Obstetric history affecting care in pregnancy
Not Related	PRG016	Previous C-section
Not Related	PRG017	Maternal care for abnormality of pelvic organs
Not Related	PRG018	Maternal care related to disorders of the placenta and placental implantation
Not Related	PRG019	Diabetes or abnormal glucose tolerance complicating pregnancy; childbirth; or the puerperium
Not Related	PRG020	Hypertension and hypertensive-related conditions complicating pregnancy; childbirth; and the puerperium
Not Related	PRG021	Maternal intrauterine infection
Not Related	PRG022	Prolonged pregnancy
Not Related	PRG023	Complications specified during childbirth
Not Related	PRG024	Malposition, disproportion or other labor complications
Not Related	PRG025	Anesthesia complications during pregnancy
Not Related	PRG026	OB-related trauma to the perineum and vulva
Not Related	PRG027	Complications specified during the puerperium
Not Related	PRG028	Other specified complications in pregnancy
Not Related	PRG029	Uncomplicated pregnancy, delivery, or puerperium
Not Related	PRG030	Maternal outcome of delivery
Not Related	RSP003	Influenza
Not Related	RSP013	Lung disease due to external agents
Not Related	RSP014	Pneumothorax
Not Related	RSP015	Mediastinal disorders
Not Related	RSP017	Postprocedural or postoperative respiratory system complication
Not Related	SKN001	Skin and subcutaneous tissue infections
Not Related	SKN002	Other specified inflammatory conditions of the skin
Not Related	SKN003	Pressure ulcer of the skin
Not Related	SKN004	Non-pressure ulcer of skin
Not Related	SKN005	Contact dermatitis
Not Related	SKN006	Postprocedural or postoperative skin complication
Not Related	SKN007	Other specified and unspecified skin disorders
Not Related	SYM001	Syncope
Not Related	SYM008	Symptoms of mental and substance use conditions
Not Related	SYM009	Abnormal findings related to substance use
Not Related	SYM010	Nervous system signs and symptoms
Not Related	SYM011	Genitourinary signs and symptoms
Not Related	SYM014	Skin/Subcutaneous signs and symptoms
Not Related	SYM015	General sensation/perception signs and symptoms
Not Related	XXX000	Code is unacceptable as a principal diagnosis PDX (only used for the inpatient default CCSR)
Not Related	XXX111	Code is unacceptable as a first-listed diagnosis DX1 (only used for the outpatient default CCSR)
Not Related	NoDX1	Only used for the default CCSR when the principal or first-listed diagnosis is missing
Not Related	InvlDX	Used to indicate the diagnosis was invalid and could not be assigned a CCSR category
